# Withaferin A Inhibits Nuclear Factor-*κ*B-Dependent Pro-Inflammatory and Stress Response Pathways in the Astrocytes

**DOI:** 10.1155/2015/381964

**Published:** 2015-07-21

**Authors:** Francesca Martorana, Giulia Guidotti, Liliana Brambilla, Daniela Rossi

**Affiliations:** Laboratory for Research on Neurodegenerative Disorders, IRCCS Fondazione Salvatore Maugeri, 27100 Pavia, Italy

## Abstract

Several lines of evidence suggest that astrocytes play a key role in modulating the immune responses of the central nervous system (CNS) to infections, injuries, or pathologies. Yet, their contribution to these processes remains mostly elusive. Astroglia are endowed with a wide range of toll-like receptors (TLR) by which they can sense infectious agents as well as endogenous danger signals released by damaged cells. Here we demonstrate that the activation of astrocytic TLR4 by bacterial lipopolysaccharide (LPS) challenge can promote nuclear factor *κ*B (NF-*κ*B)-dependent induction of pro-inflammatory and stress response mediators, particularly Tumor Necrosis Factor *α* (TNF*α*), cyclooxygenase 2 (COX-2), and inducible nitric oxide synthase (iNOS). Since the steroid lactone Withaferin A was described to inhibit NF-*κ*B activity in different cell types, we next determined the impact of this natural compound towards the identified astrocytic signalling pathway. Innate immune activation was induced by stimulation of the LPS/TLR4 axis in spinal cord astrocytes. We provide evidence that both pre-treating and post-treating the cells with Withaferin A attenuate astrocytic NF-*κ*B activity as well as the consequent production of TNF*α*, COX-2, and iNOS induced by stimulation of the LPS/TLR4 pathway. This study suggests that Withaferin A may be an eligible candidate for the treatment of neuroinflammatory and stress conditions characterized by an important astrocytic input.

## 1. Introduction

Pathologies, infections, or injuries of the central nervous system (CNS) are often accompanied by an atypical inflammatory process that occurs locally in the brain parenchyma and does not involve the peripheral immune system, with only limited leukocyte infiltration. Such a localized response is defined “neuroinflammation” and primarily involves the cellular effectors of the CNS innate immune system [1, 2]. While microglia are considered the archetypical resident innate immune cells in brain and spinal cord, there is growing evidence indicating that also astrocytes can play an active role in these processes [1–3]. Yet, their contribution has been so far much less investigated.

Astrocytes represent the major glial cell population in the CNS and constitute up to 50% of its volume. In physiological conditions, they perform various activities that are essential to maintain CNS homeostasis, being involved in synaptic transmission, brain energetics, and cerebrovascular regulation [3, 4]. However, astrocytes can also sense microbial infections through the recognition of conserved motifs expressed by a wide range of pathogens. In addition, during injury or disease, they can react to endogenous “danger signals” that are typically released by damaged cells to alert the immune system [5]. The recognition of such molecules is enabled by the astrocytic expression of specific pattern-recognition receptors, including various members of the toll-like receptor (TLR) family [1, 2, 6]. Studies* in vitro* and in human post-mortem tissues consistently reported low but detectable astrocytic expression of TLR4 [7–10], a receptor that confers cellular responsiveness to the endotoxin lipopolysaccharide (LPS), an integral component of the outer membranes of Gram-negative bacteria [11–13].

Stimulation of astrocytic TLR4 by LPS was described to trigger a signaling cascade that leads to the activation of nuclear factor *κ*B (NF-*κ*B) [14], a multifunctional transcription factor that controls the expression of several pro-inflammatory and stress response mediators [15]. Such events may be relevant in the context of chronic neurological disorders, where sustained inflammatory reactions were consistently reported to contribute to neurodegeneration and disease progression [6]. Based on this, it is reasonable to postulate that the identification of novel NF-*κ*B inhibitors that limit these effects in the astrocytes is highly desirable and may be beneficial to prevent neurodegenerative processes and to ameliorate disease outcomes.

A natural compound that was consistently reported to inhibit NF-*κ*B signaling in non-CNS cell types and pathologies is Withaferin A, a steroidal lactone isolated from the roots of* Withania somnifera* [16–21]. Despite the fact that an anti-inflammatory activity of this molecule has been reported within the CNS in microglia [22–24], its impact on astroglial cells has been poorly investigated.

In the present study, we focused on astrocytes and we specifically addressed the effects of this natural compound in preventing the astrocytic activation of NF-*κ*B, and the consequent production of potentially neurotoxic pro-inflammatory and stress response mediators.

## 2. Methods

### 2.1. Cell Cultures

The P0-17D cell line was previously generated by immortalization of neonatal mouse cortical astrocytes with the SV40 large T antigen [25]. Morphologically and biochemically, these cells resemble mature primary astrocytes [25]. P0-17D cells were routinely maintained in Dulbecco's modified Eagle's medium (DMEM, Life Technologies) supplemented with 10% fetal bovine serum (FBS, Sigma-Aldrich), 2 mM glutamine and antibiotics (Euroclone). Primary astrocyte cultures (99% GFAP-positive) were prepared from the spinal cord of newborn C57BL6/SJL mice, as previously described [26, 27]. Once the cultures reached the confluence, they were replated at the optimal density in 24-well plates with or without glass coverslips and maintained in Minimal Essential Medium (MEM, Life Technologies) supplemented with 10% FBS.

### 2.2. Transfection and Luciferase Enzymatic Assay

P0-17D cells (1 × 10^4^ cells/well) or primary astrocytes (2 × 10^4^ cells/well) were grown in 24-well plates and transiently co-transfected with the pGL4.32[*luc2P*/NF-*κ*B-RE/Hygro] reporter vector (Promega) (hereafter referred to as “NF-*κ*B-RE-*Luc* transgene”), which contains the firefly luciferase gene under the control of the NF-*κ*B-responsive elements, and the normalization plasmid pGL4.74[*hRluc*/TK] (Promega) containing the* Renilla* luciferase gene (firefly :* Renilla* vector ratio, 50 : 1). Alternatively, cells were co-transfected with the pLucTKS3 STAT3 reporter vector, which contains signal transducer and activator of transcription 3 (STAT3)-driven firefly luciferase, the* Renilla* luciferase reporter vector and the pSG5-STAT3*α* vector, a plasmid encoding the STAT3*α* protein activator. Twenty-four hours after the transfection, cells were preincubated in the absence or in the presence of increasing concentrations of Withaferin A (0.1, 0.5, 1 *μ*M; Tocris) for 1 hour and, then, stimulated with LPS (1 *μ*g/mL; Sigma-Aldrich) for another 6 hours. Cells were subsequently lysed by using a Passive Lysis Buffer (Promega) and luciferase enzymatic activity, considered as surrogate of NF-*κ*B or STAT3 transcriptional activity, was determined. Firefly and* Renilla* luciferase signals were measured using a Dual-Luciferase Reporter Assay System (Promega) according to manufacturer's instructions, and the normalized firefly luminescence/Renilla luminescence ratio was calculated per each sample.

### 2.3. Pharmacological Treatments

Pre-treatment experiments were carried out by preincubating the cells for 1 hour in the absence or in the presence of SC-514 (10 *μ*M; Selleckchem) or Withaferin A (0.5 *μ*M), followed by a stimulation with LPS (1 *μ*g/mL) for 6 hours. In post-treatment experiments, cells were exposed to LPS (1 *μ*g/mL; 6 hours). Two hours after the start of the incubation, medium was supplemented with or without Withaferin A (0.5 *μ*M) and incubation was continued for additional 4 hours. The expression levels of TNF*α*, COX-2, and iNOS mRNAs were then analyzed by quantitative reverse transcription-polymerase chain reaction (RT-qPCR) as described below.

### 2.4. Quantitative RT-PCR

The expression of TLR4, TNF*α*, COX-2, and iNOS was determined by RT-qPCR in the P0-17D cell line, primary astrocytes, or spinal cord from adult C57BL6/SJL mice, as follows. Total RNA from cell or tissue lysates was extracted using RNeasy Mini Kit (QIAGEN) according to the manufacturer's guidelines. One microgram of total RNA was reverse-transcribed using iScript cDNA Synthesis Kit according to the manufacturer's instructions (BIO-RAD). Subsequent quantitative PCR was performed with the SsoFast EvaGreen Supermix on a CFX96 Real-Time PCR Detection System (BIO-RAD). Quantification of TLR4, TNF*α*, COX-2, and iNOS mRNAs was normalized to the housekeeping gene hypoxanthine guanine phosphoribosyl transferase (HPRT) expression. The sequences of all RT-qPCR primers are listed in Table 1.

### 2.5. Immunocytochemistry

After LPS treatment, astrocytes seeded on glass coverslips were fixed in 4% paraformaldehyde for 20 minutes and subsequently immunolabelled for NF-*κ*B p65 using a rabbit polyclonal antibody (Abcam). Nuclei were stained with Hoechst 33342 (Sigma-Aldrich). Images were captured using a 40x objective lens on a DM5000 B microscope (Leica Microsystem) equipped with a digital camera DFC 310 FX (Leica Microsystem).

## 3. Results and Discussion

**Figure 1:**
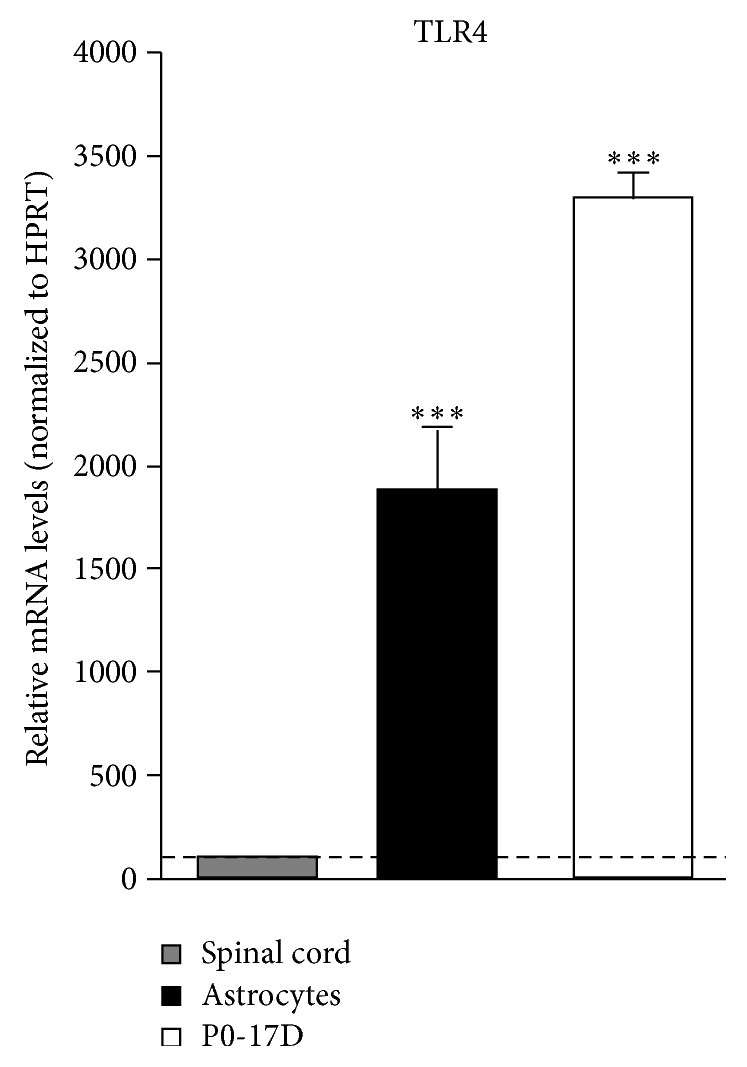
Comparative analysis of TLR4 expression in mouse spinal cord, P0-17D cell line, and primary spinal cord astrocytes. Expression of TLR4 was determined in mouse spinal cord, P0-17D cells, or primary astrocytes. Total RNA was extracted, reverse-transcribed, and analyzed by RT-qPCR (*n* = 3 in duplicate). Values (mean ± s.e.m.) were normalized relative to HPRT and expressed as percentage of TLR4 mRNA levels in mouse spinal cord (^*∗∗∗*^ *p* < 0.0001 versus mouse spinal cord; one-way ANOVA followed by Bonferroni post hoc test).

**Figure 2:**
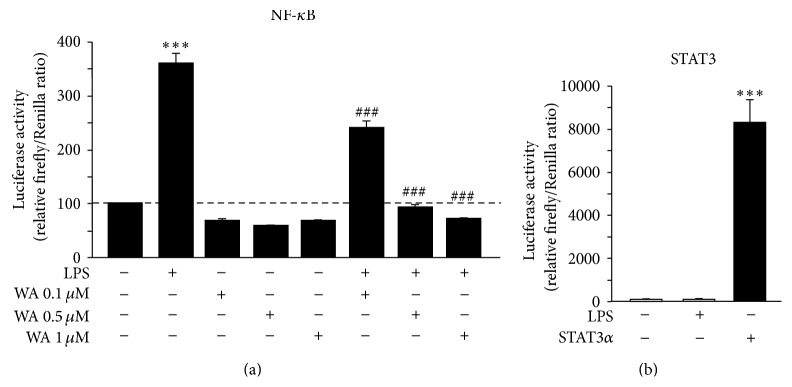
Impact of Withaferin A on LPS-induced NF-*κ*B activity in immortalized P0-17D cortical astrocytes. (a) P0-17D astrocytic cells were transiently co-transfected with the NF-*κ*B-RE-*Luc* transgene and the* Renilla* luciferase encoding plasmid, used as transfection control. Twenty-four hours after the transfection, cells were pre-treated for 1 hour with increasing concentrations of Withaferin A (WA) and then incubated in the presence or in the absence of 1 *μ*g/mL LPS for additional 6 hours (*n* = 3 experiments in triplicate). Cell lysates were assayed for luciferase enzymatic activities, and firefly luciferase levels were normalized to* Renilla* luciferase values. Data (mean ± s.e.m.) are expressed as percentage of luciferase activity in control conditions, that is, the corresponding culture type challenged with saline (^*∗∗∗*^ *p* < 0.0001 versus control, ^###^ *p* < 0.0001 versus LPS; one-way ANOVA followed by Bonferroni post hoc test). (b) P0-17D astrocytes were transiently co-transfected with STAT3-driven firefly luciferase and* Renilla* luciferase reporter vectors in the absence or in the presence of a plasmid encoding the constitutively active STAT3*α* protein. Cells were subsequently incubated with or without 1 *μ*g/mL LPS for 6 hours (*n* = 3 experiments in triplicate) and cell lysates were assayed for luciferase enzymatic activities, as above. Data (mean ± s.e.m.) are expressed as percentage of luciferase activity in control conditions, that is, the corresponding culture type challenged with saline (^*∗∗∗*^ *p* < 0.0001 versus control; one-way ANOVA followed by Bonferroni post hoc test).

To set the experimental conditions for studying the activity on astrocytes of the steroid lactone Withaferin A, we initially established a cell-based assay by using the immortalized P0-17D astrocytic cell line [25]. The potential responsiveness to LPS/TLR4 signalling of P0-17D cortical astrocytes was firstly evaluated by determining the expression of TLR4 in these cells by RT-qPCR. Quantification of TLR4-encoding transcripts confirmed significantly higher levels of the receptor-encoding mRNA in the P0-17D cells when compared to those in the mouse spinal cord (Figure 1). Since stimulation of TLR4 can trigger NF-*κ*B transcriptional activity in a vast array of cell types, we next decided to transiently transfect the P0-17D astrocytic cells with the reporter transgene NF-*κ*B-RE-*Luc*, a minimal promoter-driven firefly luciferase reporter vector containing five copies of the NF-*κ*B-responsive element. Luciferase enzymatic activity was determined, as surrogate of NF-*κ*B transcriptional activity, after treating the cells in the absence or in the presence of LPS (1 *μ*g/mL) for 6 hours. We found that LPS-induced NF-*κ*B transcriptional activity is significantly enhanced when compared with control conditions, that is, the corresponding culture type challenged with saline (Figure 2(a)). Conversely, the bacterial endotoxin was unable to trigger the transcriptional activity of the signal transducer and activator of transcription 3 (STAT3) in cells transfected with a STAT3-driven luciferase reporter vector (Figure 2(b)). The fact that LPS resulted incapable of activating STAT3 in the P0-17D astrocytic cells is at variance with previous studies describing the activation of astrocytic STAT3* in vitro* and* in vivo*, upon stimulation with the bacterial lipopolysaccharide [28–32]. Yet, all these reports determined the activation of STAT3 by detecting either the phosphorylation or the nuclear translocation of the transcription factor. The indirect nature of these approaches makes difficult to draw definitive conclusions on the actual state of STAT3 activation and transcriptional activity. By contrast, taking advantage of the luciferase reporter assay mentioned above, we could reliably measure the transcriptional activity of STAT3 in a more direct, dynamic, and quantitative fashion. Based on these results, we can conclude that, in P0-17D astrocytes, the treatment with LPS preferentially activates the NF-*κ*B transcriptional pathway, rather than STAT3 signalling.

In the next set of experiments, we exploited the P0-17D astrocyte-based luciferase reporter assay to screen the NF-*κ*B inhibitory effects of Withaferin A. Cells were pre-treated with increasing concentrations of Withaferin A (0.1, 0.5, and 1 *μ*M) for 1 hour and then incubated with LPS (1 *μ*g/mL) for additional 6 hours. As shown in Figure 2(a), LPS-induced NF-*κ*B-driven luciferase activity was significantly reduced by Withaferin A in a dose-dependent manner, reaching the maximum inhibitory effect at 0.5 *μ*M (Figure 2(a)). By contrast, Withaferin A alone did not significantly affect the basal activity of NF-*κ*B (Figure 2(a)).

**Figure 3:**
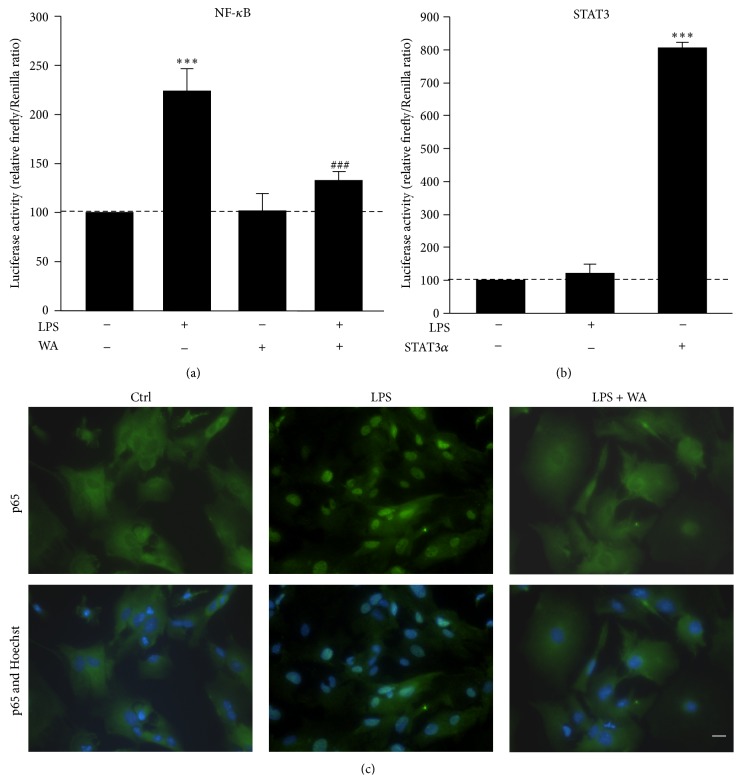
Effects of Withaferin A on LPS-induced NF-*κ*B activity in primary spinal cord astrocytes. (a) Primary astrocytes from the spinal cord of newborn mice were transiently co-transfected with NF-*κ*B-driven firefly and* Renilla* luciferase reporter vectors. Twenty-four hours after the transfection, cells were preincubated in the presence or in the absence of 0.5 *μ*M Withaferin A (WA) for 1 hour and then treated with or without 1 *μ*g/mL LPS for additional 6 hours (*n* = 4 experiments in quintuplicate). Cell lysates were assayed for luciferase enzymatic activities, and firefly luciferase levels were normalized to* Renilla* luciferase values. Data (mean ± s.e.m.) are expressed as percentage of luciferase enzymatic activity in control conditions (^*∗∗∗*^ *p* < 0.0001 versus control, ^###^ *p* < 0.0001 versus LPS; one-way ANOVA followed by Bonferroni post hoc test). (b) Primary astrocytes were transiently co-transfected with the STAT3-driven firefly and* Renilla* luciferase reporter vectors in the presence or in the absence of a plasmid encoding the STAT3*α* activator protein. Cells were subsequently incubated with or without 1 *μ*g/mL LPS for 6 hours (*n* = 3 experiments in triplicate). Cell lysates were assayed for luciferase enzymatic activities, as above. Data (mean ± s.e.m.) are expressed as percentage of luciferase activity in control conditions (^*∗∗∗*^ *p* < 0.0001 versus control; one-way ANOVA followed by Bonferroni post hoc test). (c) Astrocytic cultures were pre-treated in the absence or in the presence of 0.5 *μ*M WA for 1 hour followed by incubation with 1 *μ*g/mL LPS for 6 hours. Images are representative of cells immunolabelled for the p65 subunit of NF-*κ*B (*green*) and counterstained with Hoechst 33342 (*blue*) to visualise the nuclei. Scale bar, 20 *μ*m. Note that the treatment with LPS induces the nuclear translocation of p65 while this effect is strongly abrogated in the presence of Withaferin A.

Having established the most effective concentration of Withaferin A to inhibit NF-*κ*B, we next switched to primary spinal cord astroglial cells. Similar to P0-17D cells, we confirmed higher levels of TLR4 mRNA in primary astrocytes when compared to those in mouse spinal cord by RT-qPCR (Figure 1). The effect of Withaferin A towards LPS-induced NF-*κ*B activity was then explored in cultured spinal cord astrocytes transiently transfected with the NF-*κ*B-RE-*Luc* transgene. Cells were treated with Withaferin A (0.5 *μ*M) for 1 hour, followed by stimulation with LPS (1 *μ*g/mL, 6 hours). Using the luciferase reporter assay and immunocytochemical analysis, we could confirm the ability of this natural compound to inhibit the transcriptional activity of NF-*κ*B (Figure 3(a)) as well as the nuclear translocation of its p65 subunit (Figure 3(c)) triggered by LPS treatment. Also in this case, Withaferin A alone did not interfere with the basal activity of NF-*κ*B (Figure 3(a)). Besides, we could exclude that the effect of Withaferin A was linked to the activity of STAT3, because LPS could not activate this factor in spinal cord astrocytes (Figure 3(b)).

**Figure 4:**
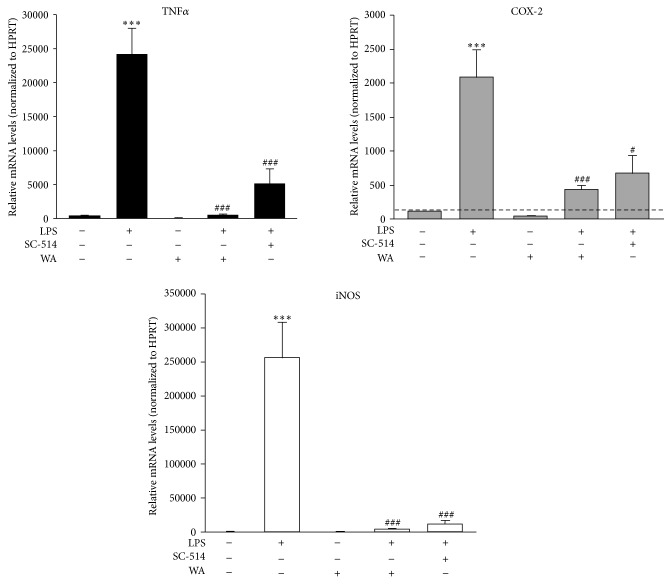
Pre-treatment with Withaferin A reduces the expression of TNF*α*, COX-2, and iNOS triggered by LPS in primary spinal cord astrocytes. Primary astrocytes were pre-treated with 10 *μ*M SC-514, a selective NF-*κ*B inhibitor, or 0.5 *μ*M Withaferin A (WA) for 1 hour and then incubated in the presence or in the absence of LPS (1 *μ*g/mL) for 6 hours. Total RNA was extracted and reverse-transcribed. TNF*α*, COX-2, and iNOS mRNA levels were analyzed by RT-qPCR (*n* = 3 experiments in triplicate). Values (mean ± s.e.m.) were normalized relative to HPRT and expressed as percentage of TNF*α*, COX-2, or iNOS expression levels in control conditions (^*∗∗∗*^ *p* < 0.0001 versus control, ^###^ *p* < 0.0001 and ^#^ *p* < 0.01 versus LPS; one-way ANOVA followed by Bonferroni post hoc test).

**Figure 5:**
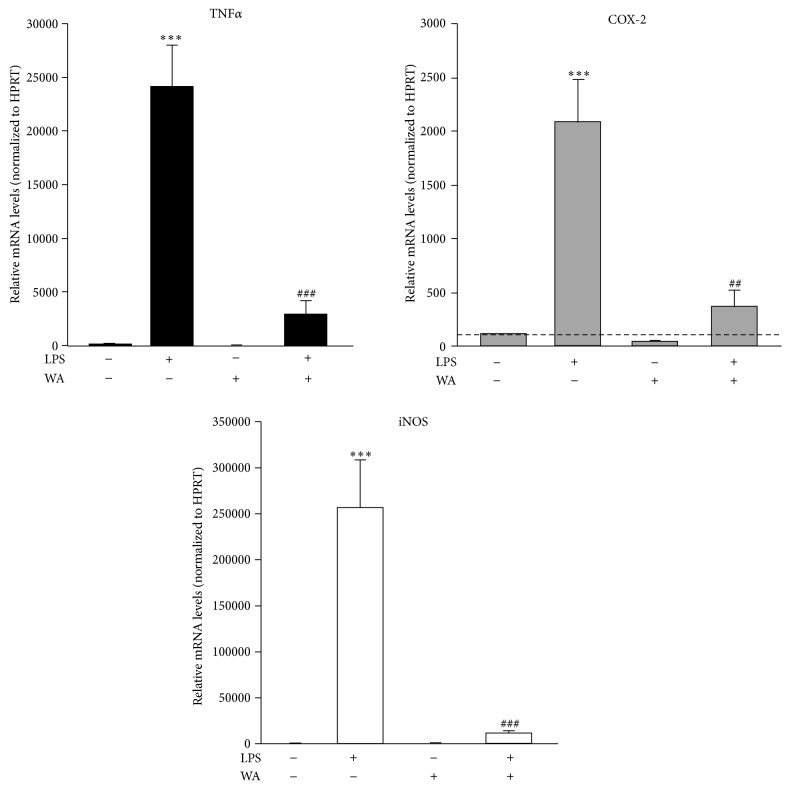
Effects of the post-treatment with Withaferin A on LPS-induced pro-inflammatory and stress response gene expression in primary spinal cord astrocytes. Primary astrocytes were treated with or without LPS (1 *μ*g/mL) for 2 hours and then incubated in the absence or in the presence of 0.5 *μ*M Withaferin A (WA) plus LPS (1 *μ*g/mL) for additional 4 hours. Total RNA was extracted and reverse-transcribed. TNF*α*, COX-2, and iNOS mRNA levels were analyzed by RT-qPCR (*n* = 2 experiments in triplicate). Values (mean ± s.e.m.) were normalized relative to HPRT and expressed as percentage of TNF*α*, COX-2, or iNOS expression levels in control conditions (^*∗∗∗*^ *p* < 0.0001 versus control, ^###^ *p* < 0.0001 and ^##^ *p* < 0.001 versus LPS; one-way ANOVA followed by Bonferroni post hoc test).

NF-*κ*B is notoriously a versatile transcription factor that controls the expression of several pro-inflammatory and stress response mediators [15]. These include, for example, the cytokine Tumor Necrosis Factor *α* (TNF*α*), the inducible isoform of the cyclooxygenase enzyme (COX-2), which is deputed to the production of eicosanoids (e.g., prostaglandins), and the inducible isoform of nitric oxide synthase (iNOS), which mediates the production of nitric oxide (NO) [33–36]. Remarkably, these proteins are widely considered key effectors of the neuroinflammatory reaction that characterizes several neurological disorders [37, 38]. In addition, considerable evidence indicates that they can contribute to excitotoxic neuronal cell death* in vitro* [39–42]. Thus, the identification of pharmacological compounds that inhibit their production is highly desirable. On these bases, we next decided to investigate the impact of LPS and Withaferin A towards the expression of these mediators in primary spinal cord astrocytes by RT-qPCR. Astroglial cells in culture were pre-treated with SC-514, a specific NF-*κ*B inhibitor, or the most effective dose of Withaferin A (0.5 *μ*M) for 1 hour and, then, exposed to LPS (1 *μ*g/mL; 6 hours). Gene expression analysis revealed that the levels of TNF*α*, COX-2, and iNOS mRNAs significantly increased after stimulation with the endotoxin (Figure 4), thus confirming the capacity of our cell model system to generate immune responses* in vitro*. In addition, we found that both SC-514 and Withaferin A significantly reduced the increment in TNF*α*, COX-2, and iNOS expression levels triggered by LPS stimulation. Given the prophylactic anti-inflammatory efficacy of Withaferin A* in vitro*, we next decided to explore the therapeutic potential of this natural compound. To this end, spinal cord astrocytes were exposed to LPS (1 *μ*g/mL) for six hours, as above. Two hours after the start of the incubation, cells were supplemented with Withaferin A (0.5 *μ*M) and incubated for additional 4 hours. RT-qPCR analysis confirmed that the post-treatment with Withaferin A is able to potently reduce the levels of mRNAs coding for TNF*α*, COX-2, and iNOS induced by LPS stimulation (Figure 5). No significant impact of Withaferin A itself on the basal expression of the three genes was detected after both the pre-treatment and the post-treatment when compared to control conditions (Figures 4 and 5).

Altogether, these data confirm that the expression of the three mediators induced by LPS is dependent on the activity of NF-*κ*B in spinal cord astrocytes. Furthermore, they highlight a role for Withaferin A in reducing the NF-*κ*B-dependent production of these neurodamaging factors (Figures 4 and 5). Although Withaferin A was previously shown to inhibit STAT3 in other cell types [22–24], we demonstrate that its anti-inflammatory effect cannot be ascribed to this mechanism, as we reveal that STAT3 activity is insensitive to the action of LPS in both P0-17D and primary spinal cord astrocytes (Figures 2(b) and 3(b)).

## 4. Conclusions

The present work shows that activation of TLR4 in spinal cord astrocytes triggers a signaling cascade leading to NF-*κ*B activation. The latter, in turn, promotes the expression of pro-inflammatory and stress response mediators, exemplified by TNF*α*, COX-2, and iNOS. These events may be relevant in the context of CNS disorders as TNF*α* itself and COX-2/iNOS metabolites, particularly prostaglandins and NO, can induce excitotoxic neuronal cell death in experimental models of neuroinflammation* in vitro* [15, 39, 40]. Furthermore, inhibition of astroglial NF-*κ*B* in vivo* by genetic approaches was reported to reduce the synthesis of inflammatory mediators in various neuropathological conditions and to prevent neurotoxic events, thus pointing at the NF-*κ*B pathway as a possible therapeutic target in various neurological diseases [43–46]. Our findings also demonstrate the effectiveness of Withaferin A in inhibiting both the transcriptional activity of NF-*κ*B and the consequent production of the pro-inflammatory and stress response mediators TNF*α*, COX-2, and iNOS in the astrocytes. Therefore, our data suggest that Withaferin A has a high therapeutic potential for the treatment of neuroinflammatory conditions characterized by a significant astrocytic contribution, and its application in models of neurodegenerative disorders deserves to be investigated.

## Figures and Tables

**Table 1:** Overview over primer sequences for RT-qPCR.

Gene	Forward primer	Reverse primer
TLR4	GGACTCTGATCATGGCACTG	CTGATCCATGCATTGGTAGGT
TNF*α*	AATGGCCTCCCTCTCATCAGTT	CCACTTGGTGGTTTGCTACGA
COX-2	AAGCGAGGACCTGGGTTCA	AAGGCGCAGTTTATGTTGTCTGT
iNOS	GTTCTCAGCCCAACAATACAAGA	GTGGACGGGTCGATGTCAC
HPRT	TGAATCACGTTTGTGTCATTA	TTCAACTTGCGCTCATCTTAG
